# First Observation of Hemoglobin G-Waimanalo and Hemoglobin Fontainebleau Cases in the Turkish Population

**DOI:** 10.4274/tjh.2015.0299

**Published:** 2016-02-17

**Authors:** Duran Canatan, Türker Bilgen, Vildan Çiftçi, Gülsüm Yazıcı, Serpil Delibaş, İbrahim Keser

**Affiliations:** 1 Antalya Genetic Diagnostic Center, Antalya, Turkey; 2 Hemoglobinopathy Diagnostic Center of Mediterranean Blood Diseases Foundation, Antalya, Turkey; 3 Namık Kemal University Research and Application Center for Scientific and Technological Investigations, Tekirdağ, Turkey; 4 Akdeniz University Faculty of Medicine, Department of Biology and Genetics, Antalya, Turkey

**Keywords:** Abnormal hemoglobins, Hemoglobin G-Waimanalo, Hemoglobin Fontainebleau

## TO THE EDITOR

Deletional alpha thalassemia mutations can be detected by various methods such as reverse dot blot, gap-polymerase chain reaction (gap-PCR), and multiplex ligation-dependent probe amplification. Point mutations leading to abnormal hemoglobins (Hb) are also observed in common populations. When a point mutation is suspected, resequencing of the alpha genes has become a routine procedure [1]. Hb G-Waimanalo is a silent mutation characterized by a substitution to aspartic acid from asparagine at codon 64 [A64(A2), Asp>Asn] [2]. Hb Fontainebleau is a slightly unstable mutation characterized by a substitution to alanine from proline at codon 21 [A21(A2), Ala>Pro] [3]. To date, they have not been reported from Turkey [4]. Here we present two cases with abnormal Hbs.

**Case 1:** NB, a 33-year-old female, was admitted to the hemoglobinopathy diagnostic center for premarital thalassemia testing. Her complete blood count (CBC) was found to be normal with 24.2% abnormal bands in high-performance liquid chromatography (HPLC) (Table 1). A blood sample was studied further at the genetic diagnostic center. Following DNA extraction with a commercial kit (Roche, Mannheim, Germany) and amplification of the whole beta globin gene by standard protocols of PCR and DNA sequencing (Applied Biosystems, USA), mutation was not found in the beta globin gene. Sequence analyses of alpha genes A1 and A2 were performed and an abnormal Hb in the HBA2: c.193G>A change was detected. This change in the HbA2 gene was at codon 64 GAC>AAC (Asp>Asn), known as Hb G-Waimanalo.

**Case 2:** ND, a 37-year-old, female, was also admitted to the hemoglobinopathy diagnostic center for premarital testing. She had normocytic anemia in CBC and abnormal bands detected at 16.2% in HPLC. This band result was found to be lower because it may have been fragmented with results of slightly unstable mutation (Table 1). Her blood was studied by the same method at the genetic diagnostic center. Mutation was not found in the beta globin gene. The HbA2 and HbA2 genes were then selectively amplified by standard protocols of PCR. DNA sequencing revealed a G to C change at nucleotide position 21 in the HbA2 gene. This mutation at codon 21 GCT>CCT (Ala>Pro) in the HbA2 gene is known as Hb Fontainebleau.

Hb G-Waimanalo is an abnormal Hb and asymptomatic. It was reported in association with alpha and/or beta thalassemia [5,6]. There were no hematological findings in our case; the beta gene was found to be normal. Hb G-Waimanalo was identified in five cases in a study in China [7].

Hb Fontainebleau was described as a silent mutation for the first time in a family of Italian origin [8]. Two cases with mild microcytosis were reported in New Zealand [3]. Our patient had normocytic anemia based on CBC and lower abnormal bands by reason of a slightly unstable mutation in HPLC. Beta gene analysis was normal. So far, a total of 22 cases including 1 homozygous case without clinical findings and 11 heterozygous cases have been reported from premarital screening in the United Arab Emirates [9].

In conclusion, abnormal bands, especially in HPLC, should be investigated with sequence analysis to corroborate alpha and/or beta globin gene mutations.

## Figures and Tables

**Table 1:**
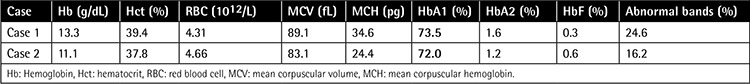
The results of complete blood count and high-performance liquid chromatography in the presented cases.
